# Tarlov cyst: a rare image

**DOI:** 10.11604/pamj.2024.48.25.43278

**Published:** 2024-05-28

**Authors:** Angan Ghosh, Sanjot Ninave

**Affiliations:** 1 Department of Anesthesia, Jawaharlal Nehru Medical College, Datta Meghe Institute of Higher Education and Research, Sawangi, India

**Keywords:** Backache, magnetic resonance imaging, cervical spine, neural foramina

## Image in medicine

A 67-year-old male came to the casualty with complaints of severe backache, inability to hold urine, and weakness in all four limbs. On neurological examination, sensory was intact and power was 3/5 in bilateral lower limbs. The patient was referred to orthopaedic department after routine examination and investigations which were normal. Magnetic resonance imaging (MRI) of the cervical spine with contrast and screening of dorsolumbar spine was done which showed subcentrimetric tarlov cyst at the neural foramina aspect of the C7/D1 disc level. A diagnosis of cervical disc disease with quadriplegia and bladder incontinence was made and the patient was posted for posterior decompression and spinal fusion.

**Figure 1:**
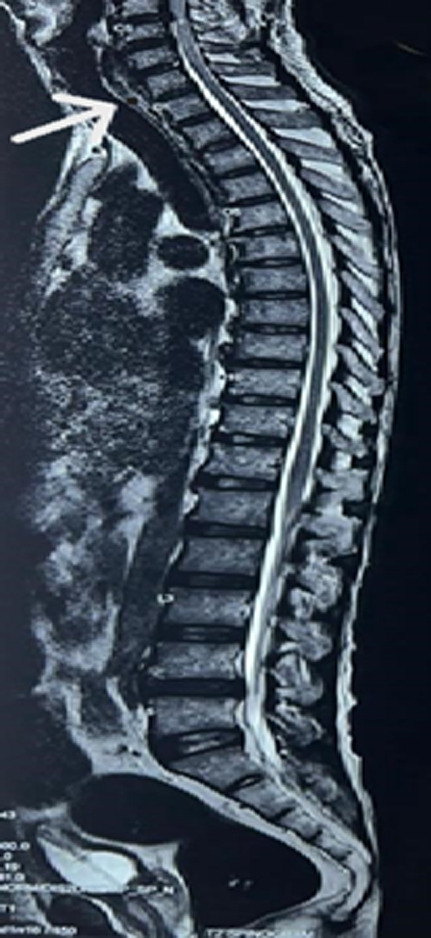
magnetic resonance imaging of the cervical spine with contrast and screening of dorsolumbar spine showing subcentrimetric tarlov cyst at neural foramina aspect of C7/D1 level

